# The Role of Premotor Areas in Dual Tasking in Healthy Controls and Persons With Multiple Sclerosis: An fNIRS Imaging Study

**DOI:** 10.3389/fnbeh.2018.00296

**Published:** 2018-12-11

**Authors:** Soha Saleh, Brian M. Sandroff, Tyler Vitiello, Oyindamola Owoeye, Armand Hoxha, Patrick Hake, Yael Goverover, Glenn Wylie, Guang Yue, John DeLuca

**Affiliations:** ^1^Human Performance and Engineering Research, Kessler Foundation, West Orange, NJ, United States; ^2^Rutgers New Jersey Medical School, Newark, NJ, United States; ^3^Department of Physical Therapy, University of Alabama at Birmingham, Birmingham, AL, United States; ^4^Department of Biomedical Engineering, New Jersey Institute of Technology, Newark, NJ, United States; ^5^Neuropsychology and Neuroscience Research, Kessler Foundation, East Hanover, NJ, United States; ^6^Department of Occupational Therapy, New York University, New York, NY, United States; ^7^Rocco Ortenzio Neuroimaging Center, Kessler Foundation, West Orange, NJ, United States

**Keywords:** multiple sclerosis, dual-task cost, fNIRS, premotor cortex, SMA, neuropsychology measures

## Abstract

Persons with multiple sclerosis (pwMS) experience declines in physical and cognitive abilities and are challenged by dual-tasks. Dual-tasking causes a drop in performance, or what is known as dual-task cost (DTC). This study examined DTC of walking speed (WS) and cognitive performance (CP) in pwMS and healthy controls (HCs) and the effect of dual-tasking on cortical activation of bilateral premotor cortices (PMC) and bilateral supplementary motor area (SMA). Fourteen pwMS and 14 HCs performed three experimental tasks: (1) single cognitive task while standing (SingCog); (2) single walking task (SingWalk); and (3) dual-task (DualT) that included concurrent performance of the SingCog and SingWalk. Six trials were collected for each condition and included measures of cortical activation, WS and CP. WS of pwMS was significantly lower than HC, but neuropsychological (NP) measures were not significantly different. pwMS and HC groups had similar DTC of WS, while DTC of CP was only significant in the MS group; processing speed and visual memory predicted 55% of this DTC. DualT vs. SingWalk recruited more right-PMC activation only in HCs and was associated with better processing speed. DualT vs. SingCog recruited more right-PMC activation and bilateral-SMA activation in both HC and pwMS. Lower baseline WS and worse processing speed measures in pwMS predicted higher recruitment of right-SMA (rSMA) activation suggesting maladaptive recruitment. Lack of significant difference in NP measures between groups does not rule out the influence of cognitive factors on dual-tasking performance and cortical activations in pwMS, which might have a negative impact on quality of life.

## Introduction

One of the hallmarks, burdensome features of multiple sclerosis (MS) involves the interrelated deterioration of both physical and cognitive performance (CP), perhaps based on co-occurring damage in neural regions that are important for those functions (Benedict et al., 2011; Motl et al., 2016; Cattaneo et al., 2017). For example, walking is a motor activity that often requires executive function and attention, especially in processing external and internal cues, so it is likely that deficits in cognitive processing contribute to gait deficits (Amboni et al., 2013). Importantly, walking in the real-word rarely occurs in isolation. That is, real-world walking is often accompanied by increased attentional demands based on performing simultaneous tasks (i.e., walking while thinking [dual-tasking] Holtzer et al., 2011). The increased attentional demands associated with dual-tasking during walking can lead to increased rate of error and consequently put persons with MS (pwMS) at an elevated risk of falling or getting injured (Wajda et al., 2013). One recent meta-analytic study reported that complex dual-tasking has negative effects on postural stability in pwMS, posing an elevated fall risk (Ghai et al., 2017). By extension, such an elevated fall risk further reduces the quality of life, ability to perform activities of daily living, and sustaining stable employment (Raggi et al., 2016). As such, quantifying the impact of dual-tasking during walking in pwMS is paramount.

Dual-tasking during walking can be measured using a variety of different paradigms. For example, paradigms that have been included in studies involving pwMS include walking while talking (e.g., alternate-letter alphabet task Learmonth et al., 2014) or mathematical calculations like subtracting by 7’s), among others to measure cognitive-motor interference (CMI). Of note, CMI is rarely expressed in terms of CP in this population, which has been acknowledged as a major limitation of the field (Leone et al., 2015; Goverover et al., 2018). Nevertheless, studies in MS samples have reported a decline in gait performance in response to adding a cognitive load vs. walking alone (Sosnoff et al., 2011; Doi et al., 2013; Learmonth et al., 2014; Downer et al., 2016). Indeed, such declines might be a product of cognitive problems associated with MS (Diamond et al., 1997; DeLuca et al., 2004a,b; Beckmann et al., 2005; Lengenfelder et al., 2006; Dobryakova et al., 2016), given that successful dual-tasking requires divided attention and the ability to process information simultaneously from multiple internal or external sources. Yet, there is equivocal evidence regarding the association between the dual-task cost of walking (DTCW; i.e., the reduction in walking performance under single- vs. dual-task conditions) and cognition in MS (Motl et al., 2014; Sosnoff et al., 2014; Kirkland et al., 2015; Sandroff et al., 2015a).

The lack of consistent associations between the DTCW and cognition in pwMS is not consistent with literature in other populations whereby the DTCW is consistently and robustly associated with aspects of information processing (Holtzer et al., 2014). Given the importance of executive functioning during walking, neuroimaging studies have focused on the role of the prefrontal cortex (PFC) during dual-tasking compared with walking alone using mobile imaging technologies like functional near-infrared spectroscopy (fNIRS), since it is not feasible to study actual walking behavior in an MRI scanner. Evidence suggests that PFC activity is elevated during dual-tasking in pwMS (Holtzer et al., 2011; Chaparro et al., 2017) as well as in healthy individuals (Mirelman et al., 2014) compared with walking alone. In addition to the PFC, premotor areas (premotor cortices (PMC) and supplementary motor area (SMA)) play an important role in executive functioning and working memory (Alvarez and Emory, 2006; Harding et al., 2015; Ptak et al., 2017), the cortical control of walking (Koenraadt et al., 2014), and in coupling cues to motor acts and in the guidance of movement (Moisa et al., 2012). Taken together, this suggests that premotor areas might play an important role in dual-tasking, which might be particularly challenged in pwMS. One recent mobile imaging study (Lu et al., 2015) reported large correlations between increased activation in these regions and a decline in gait performance while dual-tasking in healthy young adults; however, there are no published neuroimaging studies examining the roles of the PMC during dual-tasking in pwMS. Such an investigation would provide critical information for a better understanding of the neural underpinnings and potential impact of cognitive-motor interactions in this population.

The present study examined activation of bilateral premotor areas and SMA during dual-tasking (DualT), walking alone (i.e., single walking task, SingWalk) and performance of a cognitive task alone (i.e., single cognitive task, SingCog) in pwMS and healthy controls (HCs) using fNIRS. We hypothesized that relative to HCs, pwMS would demonstrate a pattern of higher premotor activation during dual-tasking relative to single tasks based on previous results from functional neuroimaging studies (Hillary et al., 2003; Rocca et al., 2009, 2012). This hypothesis is also based on the assumption that pwMS likely allocate more neural resources than HCs during dual-tasking to maximize safety by avoiding injury/falls (Sandroff et al., 2015a). We further hypothesized that in pwMS, patterns of PMC activation and behavioral DTC outcomes would be associated with measures of walking speed (WS) and neurocognitive performance.

## Materials and Methods

### Subjects

Participants with MS and HCs were recruited from local communities in NJ. Inclusion criteria for all subjects included age between 18 years and 64 years and no history of major depression, schizophrenia, bipolar disorder, or substance abuse disorders, and not taking any medications that may affect cognition and ambulation. Inclusion criteria for pwMS were: (a) clinical definite MS diagnosis (McDonald et al., 2001); (b) relapse-free for the past 30 days; and (c) ability to walk with or without a cane, but not a walker/rollator. HC participants were recruited such that each matched one of the participants with MS based on age, sex and education. A total of 270 participants were contacted, and 32 were enrolled. The final sample of participants consisted of 14 persons (2 M, 12 F) with relapsing-remitting MS and 14 age, gender and education level matched, HC participants.

### Setup and Procedure

#### Experiment Procedure

Participants were enrolled in two testing sessions, separated by a minimum of 2 days. In the 1st session, subjects were screened for eligibility and enrolled in the study after providing written informed consent approved by the Kessler Foundation Institutional Review Board, and with the 1964 Helsinki declaration and its later amendments or comparable ethical standards. Following the provision of informed consent, participants completed a set of tests to evaluate ambulation and cognition by trained personnel. Neuropsychological (NP) assessment utilized the Brief International Cognitive Assessment for MS (BICAMS; Langdon et al., 2012) battery of tests that included the following: the Symbol Digit Modalities Test (SDMT; Smith et al., 1982) as a measure of information processing speed; the second edition of California Verbal Learning Test (CVLT-II; Delis et al., 2000), as a measure of verbal learning and memory, and the Brief Visuospatial Memory Test-Revised (BVMT-R; Benedict, 1997) as a measure of visuospatial learning and memory. NP measures were administered and scored in accordance with standard published procedures (Langdon, 2015). Ambulation was measured using the timed 25-foot walk test (T25FW) based on standard procedures (Motl et al., 2017). The primary outcome from the T25FW was WS expressed as feet/second (ft/s).

Table 1 summarizes participant demographics in both groups and average of WS and BICAMS measures. T25FW WS was slower for pwMS than HC (4 ft/s vs. 4.9 ft/s, *F* = 6.01, *p* = 0.021, *d* = 0.93), while scores of BICAMS tests were not different between groups. There was also a positive correlation between WS and SDMT scores in the MS group (*r* = 0.72, *Z* = 3.01, *p* = 0.003, *r*^2^ = 0.52), indicating cognitive-motor coupling (Benedict et al., 2011).

**Table 1:** Demographics of participants in both groups.

Measure	Group	
	MS	HC
Age (years)	50 ± 8 years old	50 ± 9 years
Education (years)	15.5 ± 1.8	16.1 ± 1.6
T25FW walking speed (ft/s)*	3.99 ± 1.21	4.89 ± 0.65
BVMT-R raw score	3.14 ± 1.96	4.07 ± 2.05
CVLT-II	52.2 ± 9.26	55.86 ± 8.8
SDMT	53.2 ± 12.4	57.7 ± 5.77
WS and BVMT-R correlation	−0.15	−0.18
WS and CVLT-II correlation	0.36	−0.12
WS and SDMT correlation	0.72**	0.06

*Denotes statistically significant difference between groups (p < 0.05). **Denotes statistically significant correlation. T25FW, 25-foot walk test; SDMT, Symbol Digit Modalities Test; CVLT-II, California Verbal Learning Test; BVMT-R, Brief Visuospatial Memory Test-Revised; WS, walking speed in the T25FW test.

#### fNIRS

**Figure 1:**
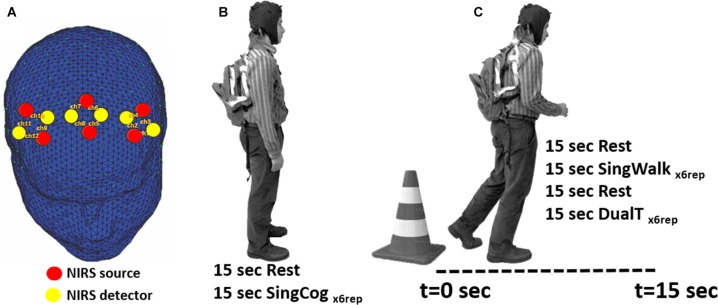
**(A)** Location of functional near-infrared spectroscopy (fNIRS) sources, detectors and channels. Location of sources and detectors are shown in red and yellow dots, respectively. The six sources and six detectors are arranged in pairs to give 12 channels that cover bilateral premotor cortices (PMC) and bilateral supplementary motor area (SMA) regions. **(B)** SingCog task, subjects were in a standing position and rested for 15 s before performing a serial 7’s mathematical calculation task for six repetitions.** (C)** Subjects were instructed to stand next to a traffic cone for 15 s; then at the Go cue, they were instructed to walk in a comfortable speed for 15 s while the researcher recorded the distance they traveled. In the SingWalk condition, they were asked to simply walk straight in a long hallway. In the DualT task, they were asked to perform the serial 7’s task as fast as they could while walking.

fNIRS technology has been successfully used in recording brain activity during ambulation (Lu et al., 2015; Chaparro et al., 2017), especially using advanced wearable multi-channel systems (Piper et al., 2014). In the present study, fNIRS (NIRSport™, NIRX, Germany) was used to collect hemodynamic activity in bilateral PMC and SMA areas. NIRSport is a wearable fNIRS system specifically designed to maximize the signal-to-noise ratio in a mobile setting (Piper et al., 2014). The optical detectors include photo-electrical receivers enclosed within a circuitry that includes a trans-impedance amplifier with a fixed 10 MΩ feedback resistor. This design provides a higher signal to noise ratio where the signal is amplified at the optode site before being transmitted to the main amplifier and data acquisition system (DAQ) and being contaminated with more motion artifacts. In the current study, six sources and six detectors were used and assembled in a montage similar to a montage used in (Lu et al., 2015) resulting in 12 total channels. Channels were arranged based on the international 10-5 system to collect data from four regions of interest (ROIs): left and right PMC (lPMC and rPMC), and left and right SMA (lSMA and rSMA; see Figure 1). NIRstar software and NIRSlab Matlab toolbox (Piper et al., 2014) were used to set up the probe locations and montages. Each of the light emitting diodes (LEDs) in this system emits dual-wavelength light (760 nm and 850 nm), and the sampling rate is 6.25 Hz.

In the second session, subjects were fitted with the fNIRS system and performed three experimental conditions: (1) single cognitive task (SingCog) while standing; (2) single walking task (SingWalk); and (3) dual-task (DualT, walking while performing the cognitive task). The cognitive task in both SingCog and DualT was a conventional paradigm that requires subtracting serial 7’s from a given number between 70 and 100, randomly chosen by the investigators (SingCog task; Leone et al., 2015), and reporting the results of subtractions verbally and aloud. Subjects carried a backpack during the SingCog, SingWalk and DualT tasks that included the data acquisition hardware of NIRsport system. The backpack weighted around 2 lb that did not result in any complaints about its weight.

Each experimental condition (SingWalk, SingCog, DualT) was repeated six times (six trials); task duration of each trial was 15 s, preceded by 15 s of rest (baseline condition) to allow the hemodynamic signal to reach a stable baseline (total 30 s × 6 trials of data per condition). The order of these conditions was randomized and counterbalanced to avoid any confounds in results related to physical or cognitive fatigue, or adaptation to the cognitive task. Behavioral measures included responses to the cognitive tasks (SingCog and DualT) as well as walking distance over the 15-s walking trials (SingWalk and DualT). Importantly, under the DualT condition, participants were explicitly asked to prioritize both walking and cognitive tasks equally.

### Data Analysis

#### Behavioral Measures

Cognitive-Motor Interference (CMI) during the walking trials was evaluated based on the change in speed (Sandroff et al., 2015a) and on the change in behavioral performance on the cognitive task across experimental conditions. DTC of WS (DTCW) was calculated as % change in WS (i.e., ((DualT − SingWalk)/(SingWalk))*100), with more negative values indicating larger reductions in WS under the DualT condition relative to the SingWalk condition. Similarly, DTC on cognitive task performance (DTCC) was calculated based on the total number of correct answers in the 15-s duration of task execution in comparison to SingCog task (i.e., ((DualT − SingCog)/(SingCog))*100).

#### Neurophysiology Measures

##### Preprocessing

fNIRS data were analyzed using NIRSlab Matlab toolbox (2017 release, Matlab2013b). Estimation of oxyhemoglobin (HbO) and deoxyhemoglobin (HbR) signals was done using Beer-Lambert Law, fitting the data to Differential Path Factor (PDF) values of 7.25 and 6.38 for 760 nm and 850 nm wavelengths, respectively (Essenpreis et al., 1993). The coefficient of variation (CV) of each channel was calculated by multiplying the standard deviation of the channel’s data by 100 and dividing it by the mean. Channels with CV >15% were considered bad channels and rejected before further processing of fNIRS data to reduce the effect of physical artifacts (Piper et al., 2014). Discontinuities and spike artifacts were removed followed by 0.01–0.14 Hz bandpass filter to exclude irrelevant frequency bands and to eliminate the effects of heartbeat, respiration and low-frequency signal drifts for each wavelength. After the rejection of channels with CV% >15, the HbO and HbR were averaged over the six trials for each condition to improve signal-to-noise ratio. Next, an average of HbO and HbR signals were calculated for the channels representing each ROI, resulting in a time series of data for four ROIs instead data of 12 channels data. The time series included data of the 15 s baseline (rest) and 15 s task for each condition. The relative changes in HbO and HbR during the 15 s tasks relative to 15 s baseline was calculated using a customized Matlab script. Then, the index of hemoglobin differential (HbDiff = Δ(HbO − HbR)) was calculated and used to evaluate brain activations in each experimental condition (i.e., SingCog, SingWalk, DualT). HbDiff was used as a parameter of cortical activation where a more positive HbO and more negative HbR results in higher HbDiff during the task period compared to baseline. An alternative to using HbDiff as a measure of brain activation was to use both ΔHbO and ΔHbR; HbDiff was chosen instead in order to simplify the analysis. This approach further has been adopted in previous fNIRS studies (Lassnigg et al., 1999; Lu et al., 2015).

##### Group Average

ANCOVA was used to compare the difference between groups in DTCC, i.e., the percent change in the number of correct answers in the DualT condition vs. SingCog, and DTCW, measured as percent change in WS in DualT condition vs. SignWalk condition. Single task behavioral data were used as covariates. A General Linear model was used to perform repeated measures Analysis of Variance (2 × 3 × 4 rANOVA) on cortical activation. Condition (SingCog, SingWalk, DualT) and ROI (bilateral PMC and SMA) were included as within-subjects factors, and group (MS vs. HC) was included as a between-subjects factor. To decompose any significant group by condition by ROI interactions from the rANOVA, we further performed follow-up 2 × 2 ANOVA comparisons between groups and (1) SingCog and DualT to understand the effect of walking in a dual-task on cortical activation; and (2) SingWalk and DualT to understand the effect of cognitive effort in a dual-task on cortical activation within ROI. Significance was set to α = 0.05 corrected for multiple comparisons when necessary. Bonferroni correction for multiple comparisons was performed using the family-wise error rate divided by the number of comparisons (c) as the significance threshold (modified α_m_ = α_FW_/c). The family-wise type I error rate was calculated assuming *α*_FW_= 1 − (1 − α)^c^ (Keppel and Wickens, 2004), So, to account for performing 2 × 2 ANOVA comparisons for the four ROIs, the *α*_FW_ was set to 0.185 and the significance threshold was set to α_m_ = 0.046.

##### Regression Analysis

Stepwise Hierarchical Regression analysis was performed to test the possible influences of baseline measures of T25FW WS and BICAMS tests scores on DTCW and DTCC. Similarly, we studied if these baseline measures predicted the change in cortical activation due to walking effort (WalkE) and cognitive effort (CogE). The change in activation due to walking effort was calculated by subtracting cortical activation in SingCog from DualT condition (WalkE = DualT − SingCog). Similarly, the change in activation due to the cognitive effort was calculated by subtracting cortical activation in SingWalk from DualT condition (CogE = DualT − SingWalk). We tested if T25FW WS or the three NP measures predicted the amplitude of these contrasts. Matlab (“stepwiselm” function) was used in this analysis, this function uses forward and backward stepwise regression to determine the final model; it uses a *p*-value of an *F*-statistic to test models with and without a potential term at each step and it stops when no single step improves the model. Similar to the ANOVA comparisons, the significance threshold was corrected for a total of 20 comparisons (eight regression tests for the eight contrasts per group and four predictors in each test), with α_m_ = α_FW_/c = 0.64/20 = 0.032.

##### Correlation Between Cortical Activation and Measures of Gait and Cognitive Performance During Dual-Tasking

Bivariate correlation analysis was used to test the relationship between cortical activations and gait performance (WS) and CP (number of answers in the serial 7’s tasks) during dual-tasks. Since a total of 8 tests were done in each group, the significance threshold was corrected similarly to the other statistical analysis tests in this manuscript with α_m_ = α_FW_/c = 0.56/16 = 0.035.

## Results

### DTCW and DTCC

**Figure 2:**
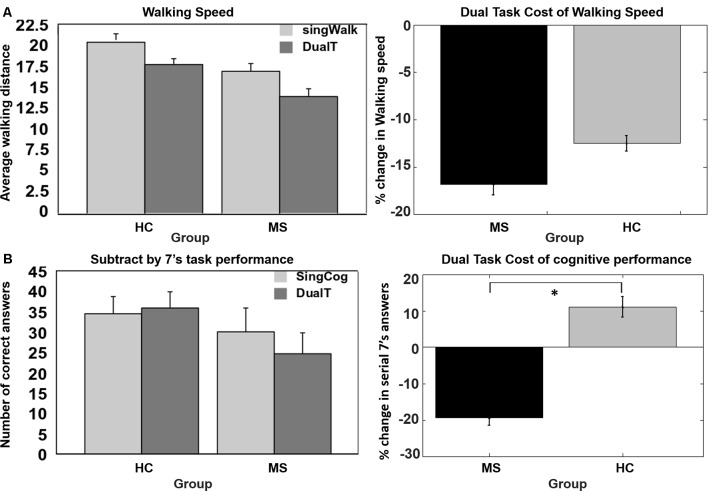
**(A)** No group difference in dual-task cost (DTC) of walking speed (WS).** (B)** Significant difference in DTC of serial 7’s cognitive task performance, decrease in performance in multiple sclerosis (MS) group and no change or improvement in performance in the healthy control (HC) group. *Denotes statistical significance (*p* < 0.05). Error bar denotes standard errors.

**Figure 3:**
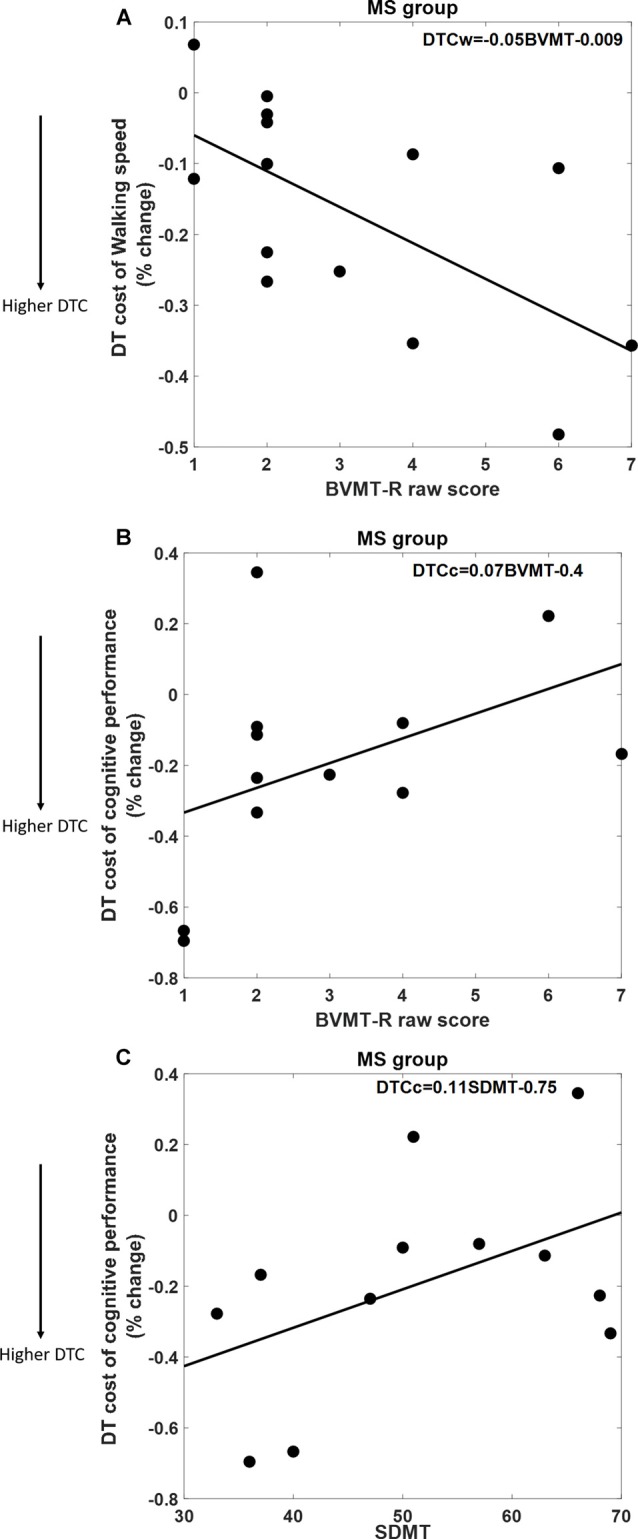
**(A)** Better Brief Visuospatial Memory Test-Revised (BVMT-R) score predicts 33% of DTC of WS. **(B,C)** Both BVMT-R and Symbol Digit Modalities Test (SDMT) scores predict 55% of DTC of cognitive performance in the serial 7’s mathematical calculations task; better BVMT-R and SDMT scores are associated with lower DTC of CP.

As shown in Figure 2, DTCW was similar in both groups; WS dropped by 16.8% (±15.7%) in the MS group, and by 12.5% (±11.3%) in the HC group. DTCC, i.e., number of serial 7’s answers, dropped by 19.3% (±30%) in the MS group and increased by 11% (±39%) in the HC group, where the HC group participants performed better under dual-task condition compared to SingCog (*F* = 5.8, *p* = 0.023, $\eta_{\text{p}}^{2}$ = 0.21). This indicates that MS had no effect on DTCW but had a significant effect on DTCC. Despite, the similar DTCW in both groups, higher DTCW (more negative values) in the MS group was predicted by a better (higher) BVMT-R score (*R*^2^ = 0.33, *F* = 5.89, *p* = 0.032, $\eta_{\text{p}}^{2}$ = 0.3). On the other hand, higher DTC of serial 7’s performance (higher deterioration in CP) in the MS group was predicted by worse BVMT-R (*F* = 5.7, *p* = 0.034, $\eta_{\text{p}}^{2}$ = 0.3) and SDMT scores (*F* = 5.3, *p* = 0.036, $\eta_{\text{p}}^{2}$ = 0.29). Together BVMT-R and SDMT explained approximately 55% of variance in DTCC (R^2^ = 0.55, *F* = 6.8, *p* = 0.01, $\eta_{\text{p}}^{2}$ = 0.55; see Figure 3). Collectively, this indicates that during the dual-task, MS participants with better information processing and visual memory focused more on the cognitive task and demonstrated higher DTC of WS and lower DTC of CP.

### Dual Task Effect on Brain Activations

**Figure 4:**
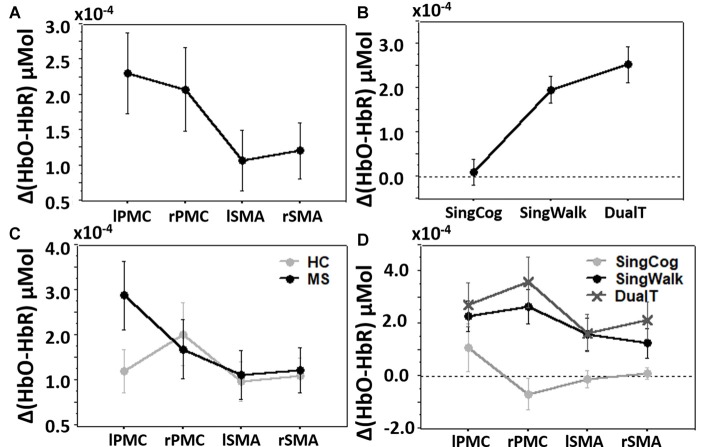
**(A)** Cortical activation in the four regions of interest (ROIs). **(B)** Cortical activation in the three conditions. **(C)** Cortical activation in the four ROIs and two groups showing ROI by group interaction driven by higher left PMC (lPMC) activation in the MS group. **(D)** Cortical activation in the four ROIs and three conditions showing ROI by condition interaction driven by higher right PMC (rPMC) and right SMA (rSMA) in the DualT condition. Error bar denotes standard errors.

Overall three-way condition by ROI by group comparison revealed significant differences between conditions (SingCog, SingWalk, DualT, *F* = 6.7, *p* = 0.003, $\eta_{\text{p}}^{2}$ = 0.34), and ROIs (lPMC, rPMC, lSMA, rSMA, *F* = 3.7, *p* = 0.016, $\eta_{\text{p}}^{2}$ = 0.22; see Figures 4A,B) and no difference between groups. There was a two-way ROI by group interaction (*F* = 2.9, *p* = 0.041, $\eta_{\text{p}}^{2}$ = 0.18) such that across conditions, left PMC activation was higher in the MS group (see Figure 4C). There was also an ROI by condition interaction such that the right PMC activation was higher in the DualT condition (*F* = 2.35, *p* = 0.033, $\eta_{\text{p}}^{2}$ = 0.15; see Figure 4D). To further understand the dual-task vs. single task effect on cortical activation in the four ROIs, we performed a 2 × 2 ANOVA to compare between groups (MS vs. HC) and DualT vs. SingWalk within each ROI. A second 2 × 2 ANOVA was done to compare between groups and DualT vs. SingCog within each ROI. The results are presented below.

### DualT vs. SingWalk

**Figure 5:**
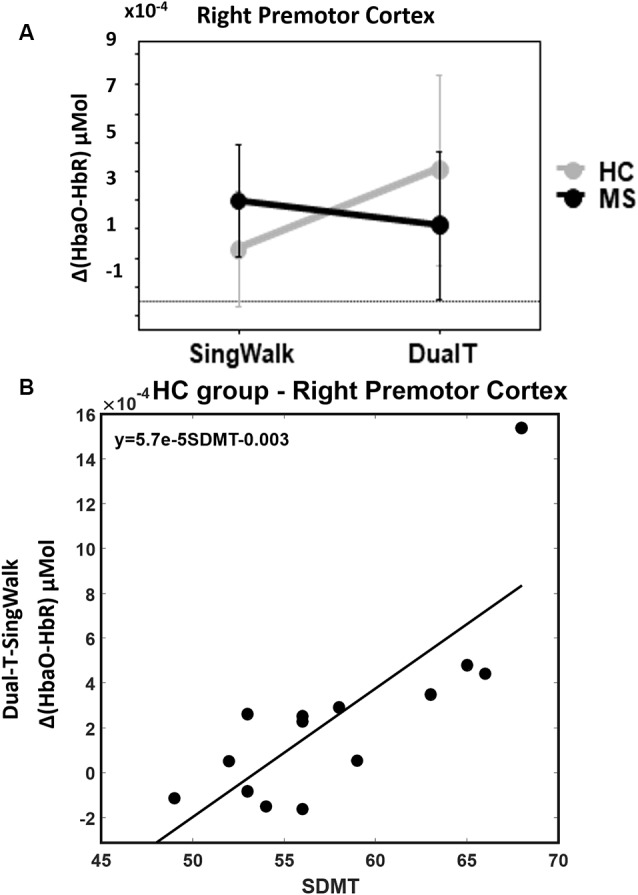
**(A)** Interaction between groups and condition (walking alone and dual-task), higher rPMC activation during dual-tasking vs. walking alone in the HC group, and no difference in the MS group. Error bar denotes standard errors.** (B)** Better SDMT scores predict higher cortical activation in dual-tasking vs. single walking task.

There was a statistically significant condition (DualT vs. SingWalk) by group (MS vs. HC) interaction (*F* = 5.1, *p* = 0.03, $\eta_{\text{p}}^{2}$ = 0.16) on rPMC activation only. *Post hoc* test within rPMC showed that HC recruited higher rPMC activation (*F* = 6.02, *p* = 0.029, $\eta_{\text{p}}^{2}$ = 0.19) under DualT conditions relative to singWalk condition while there was no change in rPMC activation in the pwMS group. The results of the regression analysis showed that the higher the information processing speed (higher SDMT score), the greater was the dual-tasking vs. single walking task effect on cortical activation in the HC group (R^2^ = 0.59, *F* = 17.3, *p* = 0.001; see Figure 5). This suggests that rPMC plays an important role in maintaining similar CP across conditions, which did not significantly change in the HC group.

### DualT vs. SingCog

**Figure 6:**
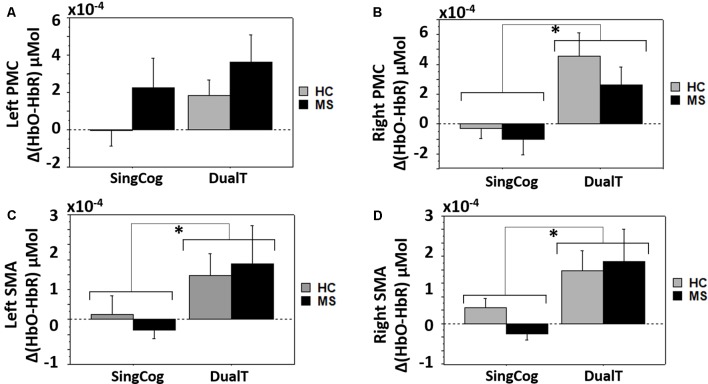
**(A)** Cortical activation in the dual-task vs. single cognitive task in lPMC in both groups. **(B–D)** Higher cortical activation in the dual-task vs. single cognitive task in rPMC, lSMA and rSMA in both groups. *Denotes statistical significance (*p* < 0.046). Error bar denotes standard errors.

**Figure 7:**
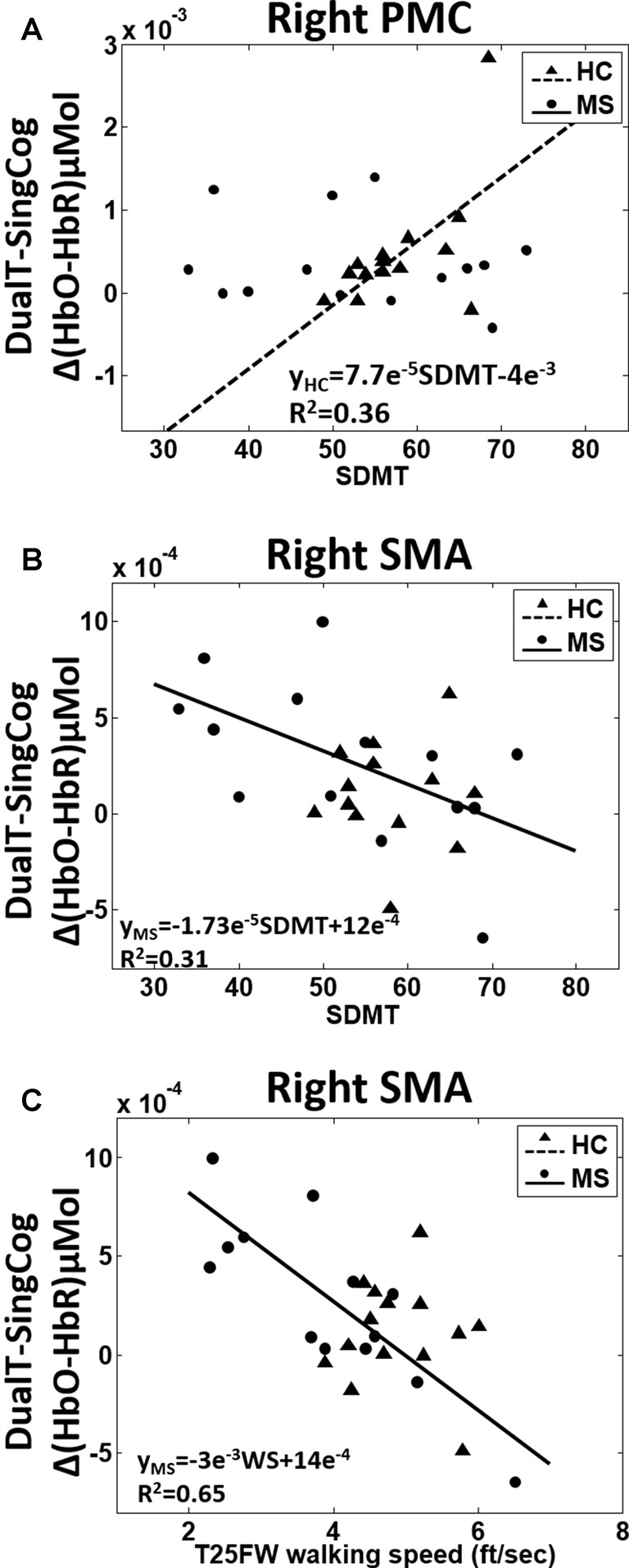
**(A)** Higher SDMT score predicts higher rPMC cortical activation in dual-tasking vs. single cognitive task in the HC group (black dotted line). **(B)** Better SDMT predicts weaker or negative difference in rSMA activation in dual-task vs. single cognitive task in the MS group (black solid line). **(C)** Faster WS predicts weaker or negative difference in rSMA activation in dual-task vs. single cognitive task in the MS group (black solid line).

There was a main effect of condition (DualT vs. SingCog) on activation in rPMC (*F* = 12.2, *p* = 0.002, $\eta_{\text{p}}^{2}$ = 0.32), lSMA (*F* = 6.5, *p* = 0.016, $\eta_{\text{p}}^{2}$ = 0.2), and rSMA (*F* = 9.5, *p* = 0.005, $\eta_{\text{p}}^{2}$ = 0.27) regions, where the activation was higher during dual-tasking in these ROIs (see Figure 6). There was no main effect of group in any ROI. However, there was a marginal effect, such that an increase in left PMC activation in the MS group with moderate effect size (lPMC; *F* = 3.7, *p* = 0.066, $\eta_{\text{p}}^{2}$ = 0.125). There was no statistically significant condition by group interaction on cortical activation in any ROI. Regression analysis showed that higher rPMC activation in DualT vs. SingCog in the HC group was predicted by better SDMT score (R^2^ = 0.36, *F* = 6.77, *p* = 0.023; see Figure 7A). Within the MS group, the effect of DualT on rPMC activation was not predicted by ambulation or NP measures. However, DualT effect on rSMA activation was associated with slower WS (R^2^ = 0.65, *F* = 22.4, *p* = 0.0005), and lower information processing speed (SDMT score; R^2^ = 0.31, *F* = 5.5, *p* = 0.037), which was marginally significant with Bonferroni correction (see Figures 7B,C).

### Relationship Between Cortical Activation and Performance During Dual-Tasking

Correlational analysis was performed to examine the relationship between cortical activation and DualT performance (see Table 2). In the MS group, lSMA and rSMA increased activation correlated with slower WS. This suggests that these regions were associated with gait adjustments during dual-tasking in pwMS. A similar relationship was not found in the HC group, and there was no significant correlation between cortical activation in the DualT and the number of answers in the serial 7’s task in the four ROIs in both groups.

**Table 2:** Correlational analysis between cortical activations and performance during dual-tasks.

Group	Condition		lPMC	rPMC	lSMA	rSMA
MS	Walking		*r* = −0.51	*r* = −0.38	*r* = −0.72*	*r* = −0.6*
	Speed		*p* = 0.06	*p* = 0.18	*p* = 0.004	*p* = 0.02
	Serial	7’s	*r* = −0.25	*r* = −0.15	*r* = −0.21	*r* = −0.28
	Answers		*p* = 0.38	*p* = 0.6	*p* = 0.46	*p* = 0.33
HC	Walking		*r* = −0.14	*r* = 0.007	*r* = −0.12	*r* = −0.29
	Speed		*p* = 0.6	*p* = 0.98	*p* = 0.68	*p* = 0.31
	Serial	7’s	*r* = 0.01	*r* = −0.1	*r* = 0.228	*r* = 0.05
	Answers		*p* = 0.74	*p* = 0.73	*p* = 0.43	*p* = 0.86

*Denotes statistically significant correlation (α_m_ < 0.035).

## Discussion

To our knowledge, this is the first examination of DTC of CP and bilateral PMC and SMA modulations of dual-tasking during dynamic tasks in pwMS vs. HC. Results showed a slowdown in speed in both groups during dual-tasking, but a deterioration in CP only in the MS group. This was observed despite the fact that the MS participants were not impaired based on traditional NP tests (i.e., BICAMS). While the MS subjects had higher DTC of CP, this was associated with the need for increased right SMA activation. This effect was greater in pwMS with lower SDMT scores (i.e., processing speed) than those with higher SDMT scores. The following sections discuss each of the primary study results within the context of how dual-tasking influences behavior and cortical activation in ambulatory and cognitive domains of functioning.

### Dual-Tasking Influence on Behavioral Performance

For both pwMS and HC groups, dual-tasking reduced WS to a similar magnitude relative to walking alone in the two groups, despite pwMS walked slower than HC in general. This finding is consistent with previous studies that have similarly reported slowing in ambulation during dual-tasking (Leone et al., 2015) relative to walking alone. The observed lack of differences in DTCW between groups is also consistent with the high-level evidence that although pwMS walk slower than HCs for both single- and dual-task walking, respectively, the DTCW is not different between persons with MS and HCs overall (Learmonth et al., 2017). By comparison, dual-tasking did affect CP (i.e., the number of correct answers in the cognitive task) relative to performing the cognitive task in isolation, where MS subjects, but not HC, performed worse in the DualT vs. SingCog condition. Despite the non-significant difference in NP (i.e., BICAMS) measures between groups, higher (better) performance in BVMT-R and SDMT correlated with lower DTCC in MS group, and higher (better) performance in BVMT-R predicted higher DTC of WS. These results suggest that individuals with MS who demonstrated better cognitive function implicitly prioritized performing the cognitive task successfully by slowing down and focusing their attention on the cognitive task.

Taken together, these findings show that even in the absence of cognitive impairment as assessed by NP testing (i.e., BICAMS), when faced with a dual-task, persons with MS can show a decline in CP. This DTC in otherwise cognitively intact persons with MS might have a negative impact on community ambulation and physical activity in pwMS (Sandroff et al., 2012, 2015b).

### Dual-Tasking Influence on Cortical Activations

Comparison of cortical activation during dual-tasking vs. SingWalk and SingCog demonstrated difference in cortical activation between the HC and MS groups. This difference in activation was in both PMC and SMA regions, and it was partially associated with cognitive function in MS group, despite similar performance as HC group on BICAMS.

#### PMC Activation

Higher rPMC activation in DualT vs. both single tasks, SingWalk and SingCog, in the HC group suggests that this cortical region is involved in both walking and cognition as single tasks, but its recruitment increases linearly with increased DTC relative to both SingWalk and SingCog. On the other hand, cortical activation did not change during the dual-tasking vs. single walking task conditions in the MS group, suggesting that the role of rPMC is altered in pwMS under these conditions. In the MS group, Group × ROI interaction showed higher left PMC activation in all the three conditions, including the SingCog task. Unlike HC, MS participants required bilateral PMC activation even to perform the single cognitive task, suggesting the need for increased recruitment to perform the cognitive task alone. This could be consistent with studies showing compensation in pwMS who are not cognitively impaired (Audoin et al., 2003; Mainero et al., 2004).

#### SMA Activation

DualT vs. SingCog task resulted in higher activation of rPMC, rSMA and lSMA regions showing no group by condition interaction. More interestingly, higher activation in rSMA in pwMS was predicted by slower WS and slower information processing speed (SDMT score). In addition, higher activation of both lSMA and rSMA during dual-tasking correlated with slower WS. SMA is a key premotor region that is involved in the control of several motor activities, including walking, so the results suggest that individuals with lower WS and SDMT score require higher activation of rSMA region to process gait performance in a dual-task vs. single cognitive task in the MS group, and to compensate for the DTC on cognition.

### Relationship Between Cortical Activation and Behavioral Performance

Interestingly, MS group showed a correlation between higher bilateral SMA activations and lower WS during dual-tasking. Similar relationship was not found in the HC group. This is inconsistent with the relationship reported in healthy young population tested in a similar dual-task experiment design (Lu et al., 2015). Lu et al. (2015) reported a correlation between higher rSMA activity and higher WS, claiming that higher brain activation contributes to maintaining gait performance. This difference in findings could be attributed to the difference in age between older HC in this study and younger HC in Lu et al. (2015) study, and it could suggest that this relationship is modulated in pwMS. More research is required to understand this relationship.

### Study Limitations

Several limitations in the design and analyses warrant acknowledgment. All the MS participants had a relapsing-remitting clinical disease course, so the present results may not generalize to participants with MS with progressive disease presentations. There is a need to explore this relationship in a larger sample that includes subjects with a wider range of MS phenotypes and impairment levels to better understand the dual-tasking effect on brain activation. Finally, although the cognitive task was relatively simple, it required delivery of quick answers within a short period of time and was selected to challenge components of information processing that are affected by MS during walking (Lengenfelder et al., 2006; Genova et al., 2012; Sandroff et al., 2014). Indeed, deficits in these aspects of information processing probably represent the primary cognitive deficit in MS (DeLuca et al., 2004a). However, a different type of cognitive task might have a different effect on brain activation (Stojanovic-Radic et al., 2014) and others (Patel et al., 2014; Kirkland et al., 2015). In addition to addressing these limitations, it is important to design dual-tasking using a non-verbal cognitive task in future studies and to investigate the role of regions in the frontoparietal network, which might be significant in processing dual-tasking and might be challenged by MS.

## Conclusion

This investigation introduces novel findings related to dual-tasking cost and the role of PMC in processing dual-tasking in pwMS and HCs. When faced with a dual-task (cognitive and motor), even MS patients with intact cognitive ability show a decline in CP. This reduction in CP while performing a dual task was associated with a different pattern of cortical activation, where left PMC was active in SingCog condition only in the MS group, and higher right SMA activation during dual-tasking correlated with worse motor and cognitive function. Further investigation is needed to delineate the roles of these regions, and other sensorimotor and fronto-parietal regions in dual-tasking, to examine the interaction between physical and cognitive tasks and the neural correlates of these behaviors, and to understand the brain mechanisms of interrelated cognitive and physical deficits in MS. Such a line of research is furtherly important for the design and implementation of targeted and optimized clinical interventions for mitigating the burden of these highly prevalent MS-related consequences (Johansson et al., 2007; Oliver et al., 2007; Motl et al., 2016).

## Author Contributions

Experiment design was performed by authors SS, BS, YG and JDL, with helpful feedback from GW and GY. Data were collected by SS, TV, OO, AH and PH. Data were analyzed by SS, OO and TV. Dissemination of findings was done by SS, BS and JDL. All authors contributed to writing the manuscript.

## Conflict of Interest Statement

The authors declare that the research was conducted in the absence of any commercial or financial relationships that could be construed as a potential conflict of interest.
